# Editorial: Health and Well-Being Related to New Family Forms: Perspectives of Adults, Couples, Children, and Professionals

**DOI:** 10.3390/ijerph20085444

**Published:** 2023-04-09

**Authors:** Dorit Segal-Engelchin, Orit Taubman-Ben-Ari

**Affiliations:** 1 The Spitzer Department of Social Work, Ben-Gurion University of the Negev, Beer-Sheva 84105, Israel; 2 School of Social Work, Bar-Ilan University, Ramat-Gan 5290002, Israel; taubman@biu.ac.il

**Keywords:** new family forms, assisted reproductive technologies, single-parent-by-choice families, same-sex parent families, donor siblings, coparenting relationships

## Abstract

The aim of this Special Issue is to advance our understanding of the factors that shape the experience, well-being, and mental health of individuals on their path to creating new family forms, including adults and children, and to inform the development of policies and practices designed to promote the thriving of these families. This Special Issue contains a collection of 13 papers that shed light on a range of micro- and macro-level factors contributing to the experience and outcomes of members of new family forms from various countries, such as the UK, Israel, Italy, China, Portugal, the Netherlands, the US, and Russia. The papers extend the current knowledge on the subject from a variety of perspectives, including medical, psychological, social, and digital communications. Their findings can aid professionals supporting members of new family forms to recognize the similarities and challenges they share with their counterparts in traditional heterosexual two-parent families, as well as their unique needs and strengths. They may also encourage policymakers to promote laws and policies designed to address the cultural, legal, and institutional constraints facing these families. Based on the overall picture that emerges from this Special Issue, we suggest valuable avenues for future research.

## 1. Introduction

In recent decades in most Western countries, the traditional family composed of a married heterosexual couple and their genetically related children has been joined by an array of new family configurations [1,2,3,4,5]. This shift is linked to the development of new reproductive technologies, as well as to major social and legal changes [6,7,8]. These include the expanding social and economic opportunities for women and their changing role in society, the greater social acceptance of sex outside of marriage, the increasing phenomenon of gay men and lesbians who are coming out of the closet, and the growing social acceptance and legal recognition of same-sex marriage and LGBTQ parent families [4,6,8,9,10].

The ever-growing diversity of new family forms encompasses, among other things, intentionally child-free families, families headed by couples choosing a living-apart-together relationship, single-parent-by-choice families, families created by elective coparenting arrangements, families headed by LGBTQ+ parents, families created via elective reproduction preservation or reproductive donation (e.g., sperm, egg, or embryo donation), and surrogacy families. This suggests that although the traditional family may not suit everyone, a high value continues to be placed on the concept of family.

The emergence of new family types has been accompanied by an increasing number of studies aimed at investigating their impact on both child and parent outcomes. Until recent years, studies on new family configurations focused largely on comparisons of parents and children in these families with their counterparts in traditional heterosexual two-parent families [7,11]. This approach reflects the commonly held view that the traditional two-parent family is the ideal model for children, and is thus regarded as the benchmark against which other family models should be assessed [1,6,12]. However, the overall picture that emerges from studies of between-group differences reveals either no differences at all, or differences that favor the parents and the children in new family forms. For example, comparisons of single mothers by choice and married mothers found no differences between the groups in mental health symptoms [13] or quality of life. Nevertheless, when controlling for economic variables and paternal involvement, the quality of life of single mothers by choice appeared to be significantly higher than that of married mothers [14]. Furthermore, single mothers by choice have been found to report higher levels of personal growth following the transition to motherhood than mothers in two-parent families [15]. Higher levels of personal growth were also reported among gay fathers who became fathers within a previous heterosexual relationship as compared to heterosexual fathers [16]. In addition, parenthood satisfaction, life satisfaction [17], and subjective well-being [18,19] have been found to be higher among gay fathers who became fathers via a variety of routes (e.g., surrogacy, shared parenting with a woman, adoption, and previous heterosexual relationship) than among heterosexual fathers.

Positive outcomes are also reported for lesbian mothers. For example, a comparison of mothers in lesbian and heterosexual two-parent families yielded no differences between the groups in psychological distress, parental distress, or well-being [20]. This is in keeping with recent findings indicating no differences in mental health symptoms between both lesbian and gay parents in adoptive two-parent families and their heterosexual counterparts [21]. Similar levels of subjective well-being and life satisfaction have also been found in comparisons of childfree-by-choice individuals, who represent another new family form that is on the rise, and those who are parents [22,23].

Similarly, studies comparing children raised in new family forms with those raised in traditional heterosexual two-parent families indicate no differences between the groups in relation to child development outcomes [7,11]. These studies have focused, among other things, on children and adolescents raised in planned lesbian and gay families [24,25,26,27,28,29], families headed by single mothers by choice [13,30], and families created via surrogacy [31,32,33]. Moreover, some studies have shown that children in new family forms exhibit better outcomes than those of children in traditional two-parent families [19,34,35,36].

More recently, research has shifted from comparative studies, in which the traditional heterosexual two-parent family serves as a control group, to those aiming at a better understanding of new family forms by delving deeper into their unique characteristics and heterogeneity. Studies have shed light on individual differences among parents and children within new family forms and the factors underlying these differences [37], with findings suggesting that family process variables, rather than family structure variables, play a key role in determining individual outcomes [11,13,21,37,38]. For example, Carone et al.’s study [39] of the psychological adjustment of children raised in gay father surrogacy families, shows that behavioral problems among these children are associated with social and family processes, such as homophobic stigmatization and negative parenting, rather than with the family structure. In a similar vein, Farr et al. [40] conducted a longitudinal study of child adjustment among lesbian, gay, and heterosexual parent adoptive families that revealed the important contribution of coparenting behaviors to children’s development over time, regardless of parental sexual orientation.

### 1.1. The Aim of the Special Issue

The aim of this Special Issue is to advance our understanding of the factors that shape the experience, well-being, and mental health of individuals on their path to creating new family forms, including adults and children, and to inform the development of policies and practices designed to promote the thriving of these families. It seeks to address the unique issues and challenges associated with new family choices both from the viewpoint of members of new families and of the professionals who are in close contact with them.

### 1.2. Overview of the Papers in the Special Issue

This Special Issue consists of 12 empirical papers and one review that highlight a wide range of factors contributing to the psychological and social outcomes of members of new family forms from various countries, such as the UK, Israel, Italy, China, Portugal, the Netherlands, the US, and Russia. This collection of papers extends current knowledge of the subject from a variety of perspectives, including medical, psychological, social, and digital communications.

Four main topics are addressed here: single-parent-by-choice families; same-sex parent families; the family lives of lesbian women; and responses to assisted reproduction technologies.

## 2. Single-Parent-by-Choice Families

Three papers deal with single-parent-by-choice families. Of these, two focus on families headed by single fathers. **Tsfati and Segal-Engelchin** [41] investigate the experience of being a single gay father by choice in Israeli society, which is known for its familistic and pronatalist values. Using intersectionality as a conceptual framework, they demonstrate the ways in which multiple dimensions of these fathers’ identities (i.e., gender, sexual orientation, relationship status, and parenthood) simultaneously intersect with macro-level social factors (i.e., social biases and discriminatory laws and policies undermining their reproductive rights) and with each other, resulting in their concurrent experiences of sense of belonging to mainstream society and sense of otherness.

In his study on Italian gay and heterosexual single fathers by choice through surrogacy, who coparent with nonparental caregivers (e.g., grandparents, babysitters, uncles/aunts), **Carone** [42] considers the impact of intergenerational transmission of coparenting on child attachment security. His findings indicate that fathers who were exposed to higher coparenting quality in their families of origin exhibit lower levels of conflictual coparenting with the nonparental caregiver, which, in turn, is associated with their children’s attachment security. The study therefore reveals the important role played by the father’s coparenting relationship in shaping child–father attachment in single-father families.

The third paper on single-parent-by-choice families focuses on mothers who used sperm donation and fathers who used egg donation and surrogacy in the UK to achieve parenthood. Based on a convergent mixed-method approach, **Jones et al.** [43] investigate these parents’ well-being and motivations for choosing lone parenthood, the sources of support they sought when they were considering the option of lone parenthood, and the level of support they received after becoming parents. Their findings show that single mothers and fathers share similar motivations for choosing lone parenthood, reach out to similar sources of support, and do not differ in their mental health or levels of perceived support. The study also highlights single fathers’ dual experience of social approval and stigma.

## 3. Same-Sex Parent Families

Three papers in this issue are devoted to same-sex parent families. Two of them consider the parents. Using Family Systems Theory as a theoretical framework, **Shenkman et al.** [44] compare the psychological welfare of gay fathers through surrogacy with that of heterosexual fathers in Israel. Their findings indicate higher levels of life satisfaction, sense of post-traumatic growth, and postnatal depressive symptomatology among gay fathers. The researchers link the higher level of postnatal depressive symptomatology reported among gay fathers to the intersection between minority stress and the stress they experienced during the COVID-19 pandemic, when the data were collected.

In their paper, **Gato et al.** [45] report on two studies designed to investigate differences in parental burnout and the balance between demands (risk factors) and resources (protection factors) among same-sex and different-sex parent families from various countries. Their studies mark the first attempt to examine parental burnout among same-sex parent families. The findings reveal that neither parental burnout nor the balance between risks and resources are associated with family type, and that the balance between risks and resources is a significant predictor of parental burnout in all families.

The third paper relating to same-sex parent families considers children raised by same-sex parents in the Netherlands, where these families enjoy a supportive legal and social environment. Using survey data based on a probability sample from Dutch population registers, **Mazrekaj et al.** [46] explore differences in behavioral outcomes between children aged 6–16 years in same-sex and different-sex parent households. No significant differences in behavioral adjustment were found. The researchers suggest that the resilience within the family systems of sexual minority families may serve as a buffer against the adverse impact of the minority stress experienced by these parents on their children’s adjustment.

## 4. Family Lives of Lesbian Women

The two papers devoted to this topic consider the family lives of lesbian women in two unique traditional cultural contexts: the ultra-Orthodox Jewish community in Israel and China.

Using social representation theory as their theoretical framework, **Ben Shlomo and Oreg** [47] explore the distinct family configurations formed by lesbian ultra-Orthodox women in Israel in order to maintain their conflicting identities as both religious and lesbian. All the women in the study had married in arranged marriages and had children. Feeling uncomfortable in their spousal relationship, they sought an alternative family model that would enable them to bridge the gap between their conflicting identities. Two alternative family models were described by the women as solutions to this dilemma. Both were formed jointly with their husbands and female partners, enabling them to simultaneously maintain an overt ultra-Orthodox identity and covert lesbian identity. In the first, the women remained married and also had a lesbian relationship with their husband’s knowledge; in the second, they divorced, but continued to conceal their lesbian relationship. The findings shed light on a silenced phenomenon in ultra-Orthodox society, revealing the costs and benefits of the two solutions that transform traditional families into non-traditional ones.

**Lo et al.** [48] focus on lesbian women in urban China, using the adult worker model as a framework to explore the challenges they encounter in their work and family lives. Their findings, based on semi-structured interviews with Chinese lesbian women and social media analysis of articles published on two Chinese online platforms, demonstrate the gender-based and sexual-orientation-based obstacles encountered by lesbian women and their ways of coping with the difficulties of concurrently participating in the family as an adult and in the work economy as a worker in China. The researchers contend that the contribution of their study goes beyond the specific case of Chinese lesbian women, as it may pave the way for future investigations of the work and family lives of other groups who live outside the traditional heterosexual family in diverse cultural contexts.

## 5. Responses to Assisted Reproductive Technologies

Experiences related to the use of assisted reproductive technologies are addressed in this issue from three different perspectives: that of physicians, donor siblings, and surrogate mothers.

**Ben-Kimhy and Taubman-Ben-Ari** [49] present findings from a qualitative study investigating the perceptions of fertility physicians assisting women undergoing egg donation. All the physicians in this innovative study believed that egg donation is an excellent solution for women with infertility problems, while ignoring the potentially negative implications of the procedure. This approach seems to protect physicians’ psychological well-being and the challenges they face on a daily basis. At the same time, however, it might hinder their ability to see the whole picture from the point of view of the women and consider the diverse aspects that impact their decision making.

In her paper on donor siblings, **Hertz** [50] examines how they navigate relationships with their half-siblings and how they situate siblinghood within the context of other relationships in their lives. Based on in-depth interviews with donor-conceived teens and young adults in the US who chose to establish contact with their donor siblings, Hertz demonstrates the three distinct stages most of them experienced—anticipation, first contact, and relationship building—and the way those stages shape individual identity formation. Her findings suggest that siblinghood among youth who share a donor requires emotional labor often taken for granted among siblings who share a household, and that these people utilize their personal knowledge of friends and family to situate their new half-siblings within their lives.

The third paper relating to the response to assisted reproduction technologies considers Russian surrogate mothers. **Yeshua-Katz and Khvorostianov** [51] examine the stigma coping strategies that surrogate mothers discuss in an online support group for surrogacy in post-Soviet Russia. Conducting a thematic analysis of the support group posts, they identified four types of coping strategies: stigma internalization; stigma avoidance; group identification; and stigma challenging. It was found that most of the posts regarding coping strategies were related to stigma avoidance strategies, such as the suggestions offered about ways to hide their pregnancy. The researchers conclude that the stigma anticipated by Russian surrogate mothers leads them to prepare for it by discussing potential coping strategies in the online group.

An additional paper presented in this Special Issue is devoted to the important task of adapting existing scales for use among sexual minorities. **Gato et al.** [52] present findings from a mixed-methods study designed to adapt and validate the Coparenting Relationship Scale—Prenatal Version (CRS-PV) in a Portuguese sample of sexual minority and heterosexual adults in a dyadic relationship who did not have children. Their findings reveal different factorial structures as a function of sexual orientation. The authors therefore stress the need to examine future coparenting relationships using different criteria for sexual minority and heterosexual individuals.

Finally, **Shenkman et al.** [53] present a review of the literature on LGBTQ parent families in Israel to advance our understanding of the experiences and challenges these families encounter within the Israeli sociocultural and legal context. Their paper, which represents the first attempt to provide an overview of the empirical literature addressing LGBTQ parent families in Israel, reveals that research on these families has only explored the experience of cisgender gay fathers and lesbian mothers, with no studies considering bisexual, transgender, or queer parents. Furthermore, the authors demonstrate the ways in which the legal and sociocultural context of Israel, including its pronatalist and familistic orientation, may shape the experience of LGBTQ parent families.

## 6. Concluding Remarks

The papers presented in this Special Issue enrich the existing body of knowledge on the emerging forms of the contemporary family by shedding light on a range of micro- and macro-level factors that shape individuals’ family choices and contribute to child and parental outcomes in specific family forms. They provide insight into the unique stressors and challenges affecting the experience of parenting, parental well-being, children’s behavioral outcomes, coparenting relationships, and siblinghood in new family forms. Moreover, they indicate new paths to such family forms, which are constantly changing and being reconceptualized, along with the growing awareness and legitimization of their existence. On the practical level, the findings reported here can aid professionals supporting members of new family forms to recognize the similarities and challenges they share with their counterparts in traditional heterosexual two-parent families, as well as their unique needs and strengths. The findings may also encourage policymakers around the world to promote laws and policies designed to address the cultural, legal, and institutional constraints facing members of new family forms.

Given the similarities in parental and child outcomes that are reported repeatedly between traditional heterosexual parent families and new family forms, it would be of benefit for future studies to expand the use of within-group designs. This could advance our understanding of the unique experiences of individuals in specific family configurations, and of the specific risks and resilience factors affecting their experience. Both members of such families and individuals who are uncertain about their family model preferences may seek professional advice from a range of professionals, such as social workers, psychologists, pediatricians, teachers, and educational consultants. Given the potential impact of their personal perceptions and beliefs on their professional advice, which may affect their clients’ decision making, further explorations of the perceptions and attitudes of these professionals towards contemporary routes to parenthood and family types are also recommended. Moreover, as online support groups are becoming increasingly important for stigmatized individuals, another valuable avenue for future research is the investigation of the usage patterns and experience in such groups among individuals in new family forms, who often encounter stigmatization and discrimination.

## Data Availability

Not applicable.
